# Feasibility and safety of impact-loading exercise in patients with multiple myeloma—a pilot study

**DOI:** 10.1007/s00520-025-09287-y

**Published:** 2025-02-28

**Authors:** Anne Kollikowski, Marei Schallock, Ruben Ringeisen, Dirk Hasenclever, Lothar Seefried, Jan-Peter Grunz, Damir Zubac, Claudia Löffler, Freerk T. Baumann, Franziska Jundt

**Affiliations:** 1 Comprehensive Cancer Center Mainfranken, University Hospital Würzburg, Josef-Schneider Straße 6, 97080 Würzburg, Germany; 2 Department of Internal Medicine II, University Hospital Würzburg, Oberdürrbacher Straße 6, 97080 Würzburg, Germany; 3 Institute for Medical Informatics, Statistics and Epidemiology, University of Leipzig, Härtelstraße 16-18, 04107 Leipzig, Germany; 4 Orthopedic Department, University of Würzburg, Brettreichstr. 11, 97074 Würzburg, Germany; 5 Department of Diagnostic and Interventional Radiology, University Hospital Würzburg, Oberdürrbacher Straße 6, 97080 Würzburg, Germany; 6 Department I of Internal Medicine, Center for Integrated Oncology Aachen Bonn Cologne Dusseldorf, University Hospital of Cologne, Kerpener Straße 62, 50937 Cologne, Germany

**Keywords:** Multiple myeloma (MM), Feasibility, Safety, Impact-loading exercise, Bone turnover marker

## Abstract

**Purpose:**

Patients with multiple myeloma (MM) develop osteolytic lesions with fractures, pain, and impaired quality of life. Preclinical data show an anabolic effect of loading exercise in osteolytic lesions of MM. This 6-month pilot study evaluated feasibility and safety of impact-loading exercise in patients with MM after assessment of spinal stability.

**Methods:**

We assigned 20 patients to perform 45 min of guided impact-loading exercise twice a week and home-based training once a week or stretching exercise twice a week. Primary endpoint was assessment of feasibility and safety. Secondary endpoints were assessments of physical performance, quality of life, and bone remineralization.

**Results:**

Of 77 eligible patients with MM, 26% accepted participation. In the impact group, 9/12 and in the stretching group 7/8 patients completed training with adherence rates of 65.8 and 81.1%. Ninety percent of the stamping and jumping exercises were performed with increasing intensity from the prescribed training volume of ≥ 100%. Low severity pain events were reported after 32.9% of impact sessions. No serious adverse events were observed. After 6 months, 6-minute walk distance increased in the impact group by 35 m and in the stretching group by 46 m, and chair-rise test improved in the stretching group by 1.7 s. Global health status increased by 24.9% in the impact group, and functional scale by 31.9% in the stretching group based upon EORTC QLQ-C30. No signs of bone remineralization were observed in computed tomography.

**Conclusion:**

Impact training is feasible and appears to be safe in selected MM patients.

**Supplementary Information:**

The online version contains supplementary material available at 10.1007/s00520-025-09287-y.

## Introduction

Multiple myeloma (MM) is the second most common hematological malignancy associated with osteolytic lesions or diffuse osteopenia leading to skeletal-related events such as pain, increased fracture risk, or spinal cord compression [1]. Skeletal-related events impair physical condition and quality of life of patients with MM [1]. Treatment of MM involves quadruplet regimens for induction and consolidation therapy, high-dose melphalan and autologous stem cell transplantation for transplant-eligible patients, and the use of bisphosphonates or denosumab to slow down the progression of osteolytic lesions and to prevent fractures [1]. However, remineralization of MM-related bone loss remains a rare event even in patients in complete remission [2]. In healthy individuals, exercise-induced mechanical signals promote muscle and bone anabolism, improve bone quality, and decrease osteoporosis [3]. Therefore, exercise might be a promising treatment option in patients with MM [4] to induce bone remineralization of osteolytic lesions.

Up to date, only seven randomized controlled trials and one retrospective review have evaluated the effects of exercise in patients with MM. Feasibility and safety have been confirmed for strength, endurance, and stretching [5–9]. In addition, effects on quality of life [5, 9], fatigue [6, 9, 10], and physical performance [5, 7–9, 11] have been demonstrated. Nicol and co-workers published results from an individualized high-intensity aerobic, resistance, and impact training program for MM patients [12]. No adverse events occurred in the study, attendance of participants to supervised training sessions was 98%, and adherence to the impact-loading exercise program was 34% [12]. Only one study has investigated the effect of physical activity in the form of whole-body vibration (WBV) exercise on bone structural and metabolic parameters in patients with the precursor condition monoclonal gammopathy of undetermined significance (MGUS) [13]. Furthermore, preclinical evidence shows that physical stimuli in the form of mechanical loading induce anabolic bone responses in the presence of aggressively growing MM cells [14]. Whether impact-loading exercise is feasible and safe in patients with MM remains largely unknown.

We report on a two-arm, 6-month pilot study with high-intensity training with jumping and impact movements and stretching training to evaluate feasibility and safety. In addition, we determine training effects on physical performance, quality of life, and bone metabolic and structural parameters.

## Material and methods

### Patient population and study design

We conducted a controlled, prospective intervention study to determine feasibility and safety of impact and stretching training in patients with MM. The study was conducted from May 2022 to March 2023 at the University Hospital of Würzburg. All participants were recruited regardless of the current concept for MM treatment or their individual treatment plan. Patients with fractures in the last 12 months or instability of the spine determined on low-dose CT were excluded from participation in the study. In addition, patients were examined for tumor-related spinal instability using the Spinal Instability Neoplastic Score (SINS) before initiating the intervention [15]. This is a validated tool that supports clinical decision-making by assessing the risk of spinal instability in patients with spinal metastases [15]. If the SINS score was 11 or higher, participants were excluded from impact but not stretching training. Two study subjects could not be assigned to the impact group after randomization due to SINS and were switched to the stretching group. Prior to any data collection, all participants gave written informed consent. The study was conducted in accordance with the Declaration of Helsinki. The protocol was also approved by the Ethics Committee of the University of Würzburg (No. 31/21-am). A biometric expert opinion on this research project was obtained in advance from the Institute for Clinical Epidemiology and Biometry at the University of Würzburg.

### Feasibility and safety

To determine feasibility and safety of impact training, we documented the training in detail and created a feasibility questionnaire. Participants completed this questionnaire every four weeks to evaluate organization of the training sessions, implementation of the training sessions including exercise load perception and potential problems or pain/discomfort, as well as the recording of well-being after the training. In addition, adherence, adverse and serious adverse events, and drop-outs were recorded.

### Assessments

Physical function tests, quality of life questionnaires, blood sampling, and whole-body, low-dose computed tomography (CT) were conducted before and after six months of exercise intervention. The 6-minute walk test and the chair-rise test were performed to determine physical performance. Quality of life was assessed using the European Organization for Research and Treatment of Cancer Quality of Life Questionnaire EORTC-QLQ-C30 and the MM focused EORTC-QLQ-MY20. A certified, automated routine clinical laboratory analyzed the following blood values: hemoglobin, calcium, creatinine, alkaline phosphatase (AP), C-terminal telopeptide of type I collagen (CTX), parathyroid hormone (PTH). Bone specific alkaline phosphatase (BAP), and N-terminal propeptide of human procollagen type I (P1NP) were assessed by the external supplier, the Medical Care Center for Laboratory Medicine and Microbiology (Würzburg, Germany). Serum receptor activator of nuclear factor kappa-Β ligand (sRANKL), osteoprotegerin (OPG), and sclerostin were assessed by Immundiagnostik AG (Bensheim, Germany). Bone structure and mineralization were assessed on whole-body, low-dose CT (Somatom Force, Siemens Healthineers, Forchheim, Germany) to identify pathologic fractures. A maximum of five representative osteolytic lesions with a diameter of ≥ 5 mm for each participant were measured (Supplementary Table 1). Signs of bone remineralization were qualitatively evaluated for the presence or absence of marginal signs of destruction, sclerosis, and trabecular signs of compaction. To compare the size of osteolytic lesions before and after exercise treatment, a sum lesion score was determined as recently described [2]. The ratio of “size after treatment” to “size before treatment” was calculated from these values and referred to as the sum lesion score. A change of the sum score of 20% was defined as clinically significant [2].

### Intervention

Impact-loading exercise was carried out for 45 min twice a week under supervision and once a week at home. The course of the training sessions was documented. Impact training consisted of stamping and jumping exercises, which were gradually increased and adapted to the resilience of each patient. New exercises or an increase in intensity (number of jumps, height or distance, and complexity of the exercise) were implemented every four weeks. Exercises increased as follows: marching on the spot, stepping up and down from a stepper, hopping on the spot or in motion, two-legged jumping, two-legged high jumping, two-legged long jumping, jumping down from a stepper, and one-legged jumping (Supplementary Table 2). Deviations from the training protocol and pain events in terms of localization and severity were documented after each training session.

Stretching exercise was performed for 45 min twice a week under supervision. At the beginning of each training session, circular and swinging movements as well as dynamic stretches were performed. Each lesson focused on the upper or lower extremities, the trunk or a combination of these areas. In two-thirds of the sessions, stretching exercises were performed for the entire body. In one-third of the sessions, progressive muscle relaxation according to Jacobsen was practiced. Relaxation was introduced at the end of the training session and applied to the arms, legs, trunk or head. Both training groups had the option of conducting the supervised training sessions live-online. In addition, training videos demonstrating the exercises were available for use at home. While utilization rates were not recorded for the stretching group, adherence, compliance, and complete execution of the exercises were documented for the impact group.

### Statistical analysis

Descriptive statistical analysis included absolute frequencies, median with interquartile range, and mean. Pairwise comparisons were calculated using the Wilcoxon signed-rank test based on small sample size and non-normally distributed data. *P<0.05 and **P<0.01 were considered significant. No adjustment for multiple testing was performed. Data was stored using Microsoft Excel. All statistical analyses were performed with the statistical software SPSS version 29.

## Results

**Fig. 1:**
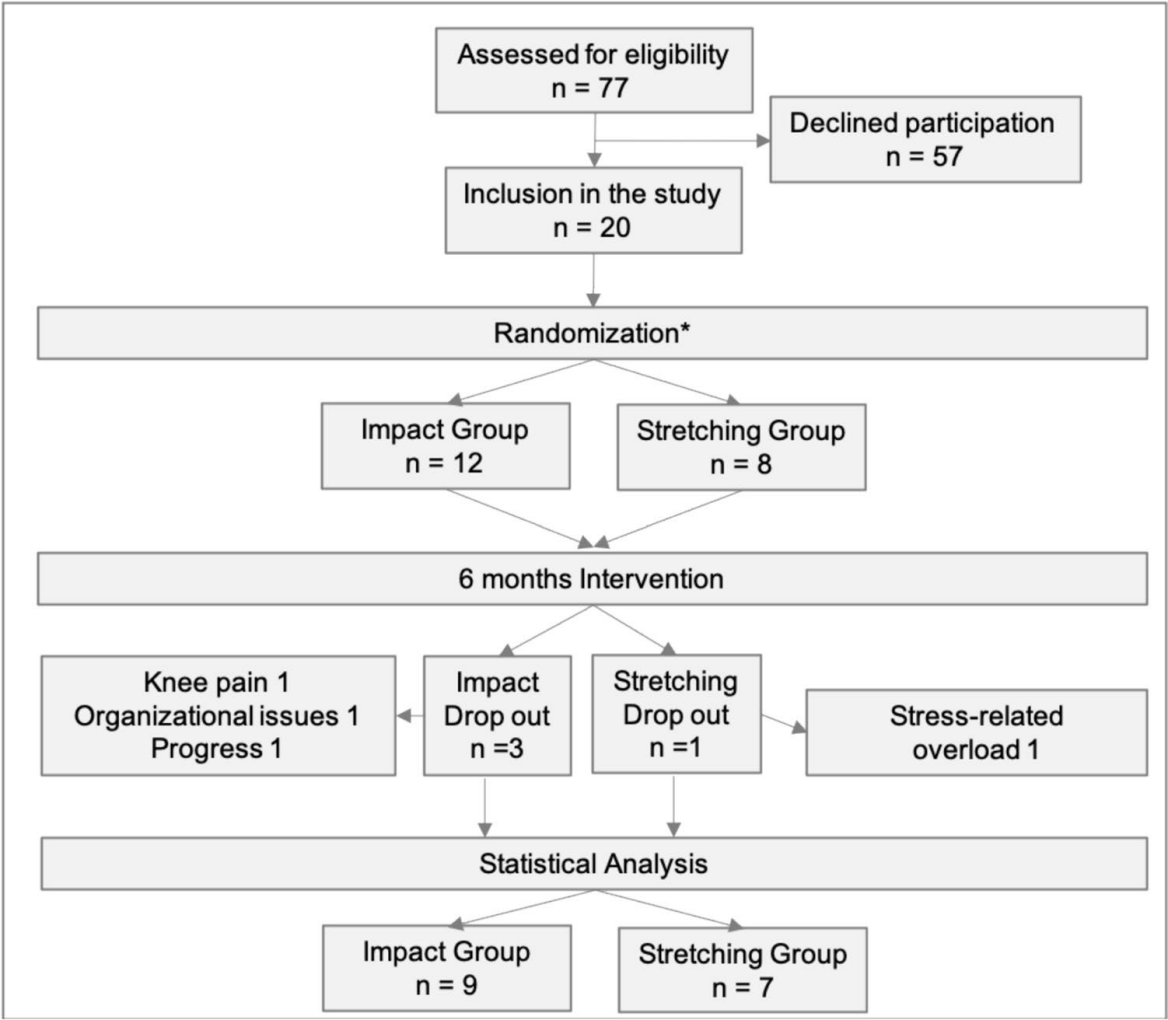
Flowchart of the study design. *Partially randomized. Two study subjects could not be assigned to the impact group and were switched to the stretching group based on SINS

Of the 77 assessed MM patients, 20 (ten males, ten females) agreed to participate in the study corresponding to a mean recruitment rate of 26% (Figure 1). Twelve patients with a mean age of 59.3 years were randomized in the impact group and eight patients with an average age of 63.1 years to the stretching group (Table 1). Two study subjects had a SINS score of 14 (ID8) and 13 (ID12) and therefore could not be assigned to the impact group (Table 1). Since the assessment of the SINS score was delayed, they were switched to the stretching group after randomization but before the start of the intervention. Fourteen out of 16 participants who completed training had osteolytic bone disease (Figure 1, Supplementary Table 1). On average, a SINS score of 6.6 and 7.3 was assigned to the participants in the impact and stretching group (Table 1). Transplant-eligible newly diagnosed patients received high-dose melphalan plus autologous stem cell transplantation for consolidation (pts. no. 4, 10) or maintenance therapy with lenalidomide (pts. no. 2, 8, 11, 15, 17). Four patients had a treatment-free interval (pts. no. 6, 9, 16, 18). For relapse therapy, regimens with daratumumab, lenalidomide or pomalidomide, bortezomib, and dexamethasone were used (Table 1). Most patients received bone treatment with zoledronic acid and vitamin D (Table 1). In the stretching group, there was one drop out due to stress-related overload, while in the impact group, there were three dropouts due to knee pain, organizational reasons, and relapse of disease (Figure 1).

**Table 1:** Characteristics of the patients: *ID*, identification; *IG*, impact group; *SG*, stretching group; *IG*, immunoglobulin; *f*, female; *m*, male; *r*, relapse therapy; *ma*, maintenance therapy; *c*, consolidation therapy; *V*, bortezomib; *D*, dexamethasone; *P*, cisplatin; *A*, doxorubicin; *C*, cyclophosphamide; *E*, etoposide; *CAR*, chimeric antigen receptor-T cell therapy. Study ID 5, 9, 12*,* and 15 are dropouts

Study ID	Impact or stretching group	IG type	Age	Gender	BMI	Pain in spine	SINS score	Current therapy	Bone treatment	Progress during the study/trial
1	IG	IgG	60	F	19	0	5	Daratumumab, pomalidomide, dexamethasone (r)	Zoledronic acid, vitamin D	No
2	IG	IgG	56	M	30	0	6	Lenalidomide (ma)	Zoledronic acid, vitamin D	No
4	IG	IgA	60	M	30	1	6	High-dose melphalan plus autologous stem cell transplantation	Vitamin D	No
5	IG	IgG	51	F	30	1	7	Daratumumab, pomalidomide - VD-PACE (r), CAR-T cell therapy (r), Teclistamab (r)	/	Yes
7	IG	IgG	59	F	27	0	6	Elotuzumab, Lenalidomide, Dexamethasone (ma)	Zoledronic acid, vitamin D	No
9	IG	Light chains	67	M	24	3	8	None	Vitamin D	Yes
10	IG	IgG	64	M	23	0	7	High-dose melphalan plus autologous stem cell transplantation; pomalidomide, bortezomib, doxorubicin and dexamethasone, daratumumab (c)	Zoledronic acid, vitamin D	No
13	IG	IgA	61	M	24	1	8	Daratumumab, bortezomib, thalidomide, dexamethasone (c), lenalidomide (ma)	Zoledronic acid, vitamin D	No
15	IG	IgG	61	F	30	0	7	Lenalidomide (ma)	Zoledronic acid, vitamin D	No
17	IG	IgA	54	M	30	1	10	Lenalidomide (ma)	Zoledronic acid, vitamin D	No
19	IG	IgG	65	F	19	1	10	Elotuzumab, carfilzomib, lenalidomide, dexamethasone (r)	Denosumab, vitamin D	No
20	IG	IgG	54	F	24	0	0	Lenalidomide (ma)	Vitamin D	No
3	SG	IgG	65	M	26	1	8	Daratumumab, lenalidomide, bortezomib, doxorubicin and dexamethasone (c), lenalidomide (ma)	/	No
6	SG	Light chains	62	M	28	0	2	None	Vitamin D	No
8	SG	IgG	69	F	25	3	14	Lenalidomide (ma)	Vitamin D	No
11	SG	IgG	62	F	29	1	8	Lenalidomide (ma)	Zoledronic acid, vitamin D	No
12	SG	Light chains	65	F	29	3	13	Daratumumab, pomalidomide, dexamethasone (r)	Zoledronic acid, vitamin D	yes
14	SG	IgG	53	M	33	0	1	None	Zoledronic acid	no
16	SG	Light chains	69	M	24	0	5	None	Vitamin D	no
18	SG	IgA	61	F	23	0	8	None	Zoledronic acid	yes

### Feasibility and safety

Participants of the impact group (*n*=9) completed an average of 34.4 out of 48 supervised, and 13 out of 24 home-based exercise sessions, corresponding to adherence rates of 71.7% and 54.2% (Supplementary Table 3). Total adherence rate was 65.8% of 72 training sessions. Reasons for missing exercise sessions were private commitments (42×) or medical reasons (70×). Participants of the stretching group (*n*=7) completed 35.7 out of 44 supervised training session, corresponding to an adherence rate of 81.1% (Supplementary Table 3). Reasons for missed appointments were private commitments (29×) or medical reasons (25×). Training in both groups was carried out in hybrid form. Participants with long distances to the study center performed supervised live-online training. In the impact group, average training duration was 45 min with an intensity of 6.63 on a scale of 1 (very light) to 10 (very hard) (Supplementary Table 3). Two hundred and seventy-eight out of 310 (89.7%) training sessions were performed at a dose level of 100% or more according to the protocol (Table 2). Perceived exertion of the load was 5.8 (Table 2). In 143 of 310 (46.1%) training sessions, the protocol was intensified, while in 32 (10.3%) training sessions, the training had to be reduced due to overload or discomfort (Supplementary Table 3). Pain events in the impact group were reported in one-third of the training sessions (102 sessions, 32.9%, Table 2). Pain was reported in 65.7% immediately after training in training weeks 5–8 (19.6%), 17–20 (26.5%), and 21–24 (24.5%) (Table 2). Severity of the pain was classified on a scale from 1 (very slight) to 10 (very severe) with an average score of 3 and occurred in the sacroiliac joint (27×), spine (25×), and knee (25×) (Table 2). In the stretching group, training duration was 45 min. On a scale of 1 (very poor) to 10 (very good), the participants rated their well-being during training as 7.8 on average (Supplementary Table 3). No pain events were recorded. No serious adverse advents were reported for both groups.

**Table 2:** Prescribed training volume and pain events recorded during impact-loading exercise. *n.r*., not recorded

Study ID	1	2	4	7	10	13	17	19	20	Total
Total number of exercise sessions	40	47	50	42	48	63	54	51	32	427
Supervised exercise sessions	30	31	35	30	36	39	46	41	22	310
Number of supervised exercise sessions from the prescribed training volume ≥ 100%	30	31	35	30	36	39	45	11	21	278 (89.7%)
Number of reported pain events during supervised exercise sessions	0	2	34	1	9	16	0	38	2	102 (32.9)
Pain events before exercise	-	-	1	-	1	-	-	-	-	2
Pain events immediately after exercise	-	1	19	1	7	13	-	24	2	67
Pain events 2–3 days after exercise	-	1	1	-	2	1	-	5	-	10
Missing data	-	-	13	-	-	2	-	9	-	24
Weeks 1–4	-	1	3	-	-	-	-	5	-	9
Weeks 5–8	-	-	8	1	2	3	-	4	2	20
Weeks 9–12	-	-	-	-	-	1	-	7	-	8
Weeks 13–16	-	-	4	-	-	3	-	6	-	13
Weeks 17–20	-	-	8	-	3	6	-	10	-	27
Weeks 21–24	-	1	11	-	4	3	-	6		25
Perceived exercise intensity (scale 1–10)	-	5.5	4.5	7	4.8	6.5	-	5.9	6.5	Ø 5.8
Localization of pain	-	Knee (1)	Spine (15), Knee (14), ISG joint (3), Hip (3)	Knee (1)	Knee (9)	Spine (10), n.r. (6)	-	ISG joint (24), muscle pain (8), thigh neck (3), spine (1), n.r. (2)	Ankle joint (2)	
Pain intensity on average (1–3 low/ 4–7 moderate / 8–10 high)	-	1.5	2.8	3	3.4	2.9	-	4.3	3	Ø 3
Tumor-associated pain	No	No	No	No	No	Yes, spine	No	No	No	

According to the feasibility questionnaire, both groups found it easy to organize appointments. Most organizational effort was involved in the journey. Participants stated that they felt the training sessions tended to be too short, but that the exercises were well explained and easy to carry out. Information on pain during and immediately after the training session is congruent with the training documentation. After training, patients stated that they felt very well. Again, the information on pain is congruent with the training documentation (Supplementary Table 4).

### Quality of life and disease-specific symptoms

Quality of life was assessed using EORTC QLQ-C30 and EORTC QLQ-MY20 questionnaires (Table 3). Global health status improved in both groups. The functional scale showed a higher score (8.9 points; *P*=0.018), and the symptom scale a lower score (12.5 points; *P*=0.042) in the stretching group (Table 3). Detailed results from both questionnaires are shown in Supplementary Tables 5–7.

**Table 3:** Results of the quality of life assessment using the EORTC QLQ-C30 and the disease-specific questionnaire EORTC QLQ-MY20, median [interquartile range]. *nominal *P*<0.05 without correction for testing multiple endpoints

Group		Scales	Baseline	6 months	Absolute change	*P* value
Impact group (*n*=9)	C30	Global health status	66.7 [37.5; 79.2]	83.3 [58.3; 95.8]	16.7 [4.2; 29.2]	0.020*
		Functional scale	86.7 [63.3; 88.9]	80.0 [60.0; 87.8]	−2.2 [−12.2; 3.3]	0.398
	MY20	Functional scales	58.3 [45.8; 70.8]	58.3 [37.5; 66.7]	0.0 [−16.7; 4.2]	0.196
		Symptom scales	25,0 [12.2; 43.3]	29,2 [10.6; 54.2]	0.0 [−10.0; 11.2]	0.779
Stretching group (*n*=7)	C30	Global health status	50.0 [41.7; 58.3]	66.7 [50.0; 75.0]	8.3 [0.0; 25.0]	0.140
		Functional scale	58.9 [46.7; 64.4]	77.7 [53.3; 80.0]	8.9 [4.4; 18.9]	0.018*
	MY20	Functional scales	66.7 [41.7; 91.7]	58.3 [50.0; 100.0]	8.3 [−16.7; 8.3]	0.729
		Symptom scales	26.6 [15.5; 25.8]	16.7 [12.5; 27.0]	−12.5 [−14.1; −3.9]	0.042*

### Physical performance

Physical assessment of the impact group showed a longer walking distance as evidenced by the 6-minute walk test (Table 4). Mean distance between baseline and after six months increased by 35 m (*P*=0.033*), while chair-rise test did not reveal changes (Table 4). Participants of the stretching group demonstrated an increase in the 6-minute walking distance from 460 to 532 m after six months. In addition, the time to rise from a chair was shorter after six months with 7.2 s compared to 8.9 at baseline (*P*=0.018*).

**Table 4:** Parameters of physical assessment at baseline and 6 months, Wilcoxon test, median [interquartile range]. *nominal *P*<0.05 without correction for testing multiple endpoints

Group	Physical assessment	Baseline	6 months	Absolute change	*P* value
Impact (*n*=9)	6-minute walk test [m]	574 [554; 624]	640 [571; 666]	35 [5; 68]	0.033*
	Chair-rise test [s]	8.0 [6.6; 8.4]	8.1 [6.7; 8.6]	0.4 [−0.9; 1.0]	0.512
Stretching (*n*=7)	6-minute walk test [m]	460 [304; 570]	532 [350; 623]	46 [0; 84]	0.075
	Chair-rise test [s]	8.9 [7.4; 17.3]	7.2 [6.3; 16.8]	−1.7 [−6.1; −0.5]	0.018*

### Bone turnover marker and bone structure

Blood tests were performed at baseline and after six months of training (Table 5). In both groups, levels of AP (IG, 80 to 17.5 ng/l, *P*=0.004; SG, 71 to 18.5 U/l, *P*=0.016), P1NP, sRANKL, and the RANKL/OPG ratio decreased, while levels of PTH increased (IG 41 to 65.6 ng/l, *P*=0.039; SG, 24 to 41.9 ng/l, *P*=0.031) after six months. All other parameters including hemoglobin, calcium, and creatinine levels remained unchanged (Supplementary Table 8). Vitamin D levels fell slightly in the stretching group depending on the season (Supplementary Table 8).

**Table 5:** Serum levels of bone turnover markers: Median [interquartile range]; *AP*, alkaline phosphatase; *P1NP*, N-terminal propeptide of human procollagen type I; *sRANKL*, serum receptor activator of nuclear factor kappa-Β ligand; *OPG*, osteoprotegerin; *CTX*, C-terminal telopeptide of type I collagen; *BAP*, bone specific alkaline phosphatase; *PTH*, sclerostin, parathyroid hormone; *m*, male; *f*, female. *nominal *P*<0.05 without correction for testing multiple endpoints

			Impact group (*n*=9)				Stretching group (*n*=7)			
	Variable	Normal range	Baseline	6 months	Change in %	*P* value	Baseline	6 months	Change in %	*P* value
Decrease of values	AP [U/l]	35–105	80 [70.5; 110]	17.5 [10; 38.05]	−78.1	0.004**	71 [53; 73]	18.5 [18.1; 25.6]	−73.9	0.016*
	P1NP [µg/l]	13.9–85.5 (m), 20.3–76.3 (f)	37.5 [16.05; 73.05]	24.3 [22.5; 69.7]	−35.2	0.82	41 [27.5; 59.4]	37.6 [24; 52.2]	−8.3	0.031*
	RANKL/OPG		142.91 [7.23; 784.35]	36.22 [6.22; 462.57]	−74.7	0.164	24.93 [2.12; 49.13]	15.53 [1.46; 225.61]	−37.7	0.813
	sRANKL [pmol/l]	90–1526 (m), 60–3400 (f)	528.48 [33.03; 3304.74]	244.6 [27.48; 2688.87]	−53.7	0.547	96 [11.29; 271.99]	67 [13.79; 908.76]	−30	0.938
Increase of values	OPG [pmol/l]	4.05 ± 1.146 (m), 6.10 ± 2.392 (f)	4.04 [3.83; 4.94]	4.58 [3.83;5.61]	+13.3	0.203	4.54 [4.15; 6.27]	5.16 [4.34; 6.17]	+13.5	0.219
	CTX [ng/ml]	0.034–1.037	0.03 [0.03; 0.27]	0.04 [0.03; 0.19]	+33.3	1	0.1 [0.05; 0.31]	0.11 [0.03; 0.37]	+10	0.313
	Sclerostin [pmol/l]	27.54 ± 14.23	31.34 [20.88; 44.87]	35.44 [21.23; 42.49]	+13.1	0.91	40.7 [30.71; 46.88]	39.98 [32.49; 46.09]	−1.8	0.156
	BAP [µg/l]	5.5–22.9 (m), 5.2–24.4 (f)	9.4 [6.9; 16.35]	10.3 [6.65; 18.4]	+9.6	0.496	7.8 [5.8; 13.5]	8.2 [6.3; 10.7]	+5.1	0.656
	PTH [ng/l]	15–65	41 [21.6; 48]	65.6 [42.85; 76.05]	+60	0.039*	24 [17.7; 45.6]	41.9 [31.5; 71]	+74.6	0.031*

Next, we performed low-dose CT in all patients before and after six months of training (Supplementary Table 1). Lesions with a minimum size of 5 mm were detected in seven of the nine patients in the impact group and in all seven patients in the stretching group (Supplementary Table 1). A total of 56 lesions, 30 (54%) in the impact group and 26 (46%) in the stretching group, were analyzed, and the sum lesion score was determined at baseline and after six months as previously described. No differences of the sum lesion score or fractures were observed after the 6-month period (Supplementary Table 1). One patient showed a post-interventional increase in lesion size of 288 mm^2^ (34%) due to disease progression.

## Discussion

We conducted a two-arm 6-month pilot study, showing that high-intensity training with jumping and impact movements was feasible and safe in selected patients with MM. No serious adverse events occurred, despite the fact that the majority of participants had osteolytic bone lesions. Pain events of low severity were reported in one-third of the impact-loading sessions immediately after training. Similar results were found in a study published shortly before the end of our project. An individualized exercise program for patients with MM who received a combination of endurance, strength, and impact training was found to be safe, feasible, and acceptable [12]. Supervised exercise training took place twice a week for 12 weeks in this study [12]. Results of our and the published study were comparable with respect to the number of repetitions of impacts (160 to 165) applied to patients. In 66.5% of the sessions, participants were not able to adhere to the specified number and intensity of impacts in the previous study [12]. In our study, participants failed to adhere to the number and intensity of impacts in only 10.3%, while the required number of repetitions was achieved or even exceeded in 89.7% of the supervised exercise sessions. One possible explanation is that patients were younger (59.3 vs 65.0, [12]) and in better physical condition in the impact group in our study. Self-reported pain events with low mean intensity of one (scale: 0–8; [12]) and three (scale: 0–10) were comparable between the studies.

We further assessed parameters of quality of life. Global health status improved in both groups as evidenced by the EORTC QLQ-C30 questionnaire. Due to the lower number of participants in the stretching group, significance was not reached. In the stretching group, the functional scale (*P*=0.18) including physical and role functioning showed higher values over time. Concomitantly, symptom scales revealed lower values for the stretching group in the EORTC QLQ-MY20 questionnaire, indicating that symptoms of fatigue, pain, and dyspnea were ameliorated. We assume that the low-threshold exercise and relaxation part of the stretching group might have led to a greater sense of well-being. In an observational study, feasibility and exercise adherence, efficacy, and quality of life of MM patients who received oncological exercise and training therapy (OTT) were examined [9]. Training included strength and endurance exercises [9]. Only one cohort showed a significant result for the functional scale (*P*=0.022) and a positive trend for the fatigue scale (*P*=0.078) from the EORTC QLQ-C30 questionnaire [9]. Similarly, in a single-arm study with MGUS and smoldering MM, patients who received aerobic and resistance training, physical functioning scores and fatigue from the functional assessment of chronic illness therapy (FACIT)-fatigue questionnaire improved [5].

In healthy individuals and in patients with osteoporosis, exercise promotes muscle and bone anabolism [3]. To evaluate whether exercise had the potential to improve physical functioning parameters in patients with MM, we determined the 6-minute walking distance and performed chair-rise tests. The 6-minute walking distance increased with both training interventions after six months. In addition, the chair-rise test improved in the stretching group. Our data indicate that six months of impact-loading or stretching exercise were sufficient to induce anabolic responses of specific muscle groups. In line with this data, we showed that patients with MGUS had an improvement in the 6-minute walk test after six months of WBV exercise [13]. In addition, another pilot feasibility study investigating the effects of strength and walking training interventions also led to an improvement in the 6-minute walk test in patients with MM after six months [7].

To evaluate a potential bone anabolic response, we determined serum levels of bone turnover markers and measured osteolytic lesion size in CT scans of participants at baseline and after six months. Levels of AP, P1NP, sRANKL, and the RANKL/OPG decreased over time. Since these changes of bone turnover markers are characteristic of antiresorptive treatment with bisphosphonates coincidentally applied in almost all patients [16–18], it remains questionable to what extent changes can be attributed to the interventions. However, these results are in line with our previous observation that WBV exercise in patients with MGUS induced a similar change in bone turnover markers and decreased levels of AP and P1NP [13]. Patients in this study did not routinely receive antiresorptive treatment suggesting that the exercise intervention contributed to the decreased levels of both markers. In parallel, PTH levels increased in the impact and stretching groups after six months. Similar changes in PTH levels after exercise have been documented in previous exercise studies [19, 20]. Lombardi et al. reported that physical activity, particularly in the late phase of prolonged exercise and during the recovery phase, led to increased PTH levels if the activity reached a specific threshold of intensity and/or duration [19]. In a preclinical study, Gardinier et al. showed that PTH induced osteogenic adaptation following exercise [20]. Thus, it was shown that PTH signaling during moderate running exercise not only increased trabecular bone formation through plate-like formation in the murine tibial region, but also improved the structural mechanical properties of cortical bone [20]. Although we observed an effect on specific bone turnover markers, we were not able to detect an anabolic effect on mineralized bone structures, possibly because the intervention period was too short, and changes in bone structures only occur after longer training periods.

Due to heterogeneity of the collective and pilot nature of the study with resulting small number of cases, it is not possible to precisely categorize our observations regarding physical performance, quality of life, and bone remodeling or remineralization. As with most exercise studies published to date, our study also shows a large number of patients who need to be approached in order to participate in such exercise studies, as only one in four patients agrees to take part. In addition, a drop-out rate of 20% must be expected over a course of six months. Impact-loading exercise generally requires supervision and close support. For this reason, it cannot be easily transferred to home-based use or to larger groups of patients. Confirmatory follow-up studies should now follow once feasibility of impact-loading exercise and preliminary safety data have been demonstrated.

## Conclusions

Impact-loading exercise is feasible and appears to be safe in selected MM patients with osteolytic lesions regardless of current treatment. Barriers for participation such as long travel to the study center were overcome by offering supervised live-online training, confirming feasibility and safety of this intervention. Although the number of participants in both study groups was small, the training interventions showed a trend for an improvement in physical performance and quality of life.

## Supplementary Information

Below is the link to the electronic supplementary material.

Supplementary file 1 (PDF 150 KB)

## Data Availability

The data sets generated and/or analyzed during this study are available on request.
