# Supplementary material for: Feasibility and safety of impact-loading exercise in patients with multiple myeloma—a pilot study
Source: Support Care Cancer. 2025 Feb 28;33(3):235. doi: 10.1007/s00520-025-09287-y (PMC11870939; doi:10.1007/s00520-025-09287-y)
Supplement: Supplementary file 1 — (PDF 150 KB) [file 520_2025_9287_MOESM1_ESM.pdf]

Supplementary information

Feasibility and safety of impact training in patients with multiple myeloma – a pilot study

Anne Kollikowski, Marei Schallock, Ruben Ringeisen, Dirk Hasenclever, Lothar Seefried, Jan-Peter Grunz, Damir Zubac, Claudia Löffler, Freerk T. Baumann, Franziska Jundt

- Supplementary Table 1
- Supplementary Table 2
- Supplementary Table 3
- Supplementary Table 4
- Supplementary Table 5
- Supplementary Table 6
- Supplementary Table 7
- Supplementary Table 8

Study ID	Impact or Stretching Group	Number of Lesions	Sum of Lesion Sizes at Baseline [mm^2]	Sum of Lesion Sizes after Treatment [mm^2]	Difference [mm^2]	Sum of Response Ratio	Mean Response [%]
1	IG	5	899.5	893.5	-6	0.99	-1%
2	IG	5	1176.5	1171.5	-5	1.00	0%
7	IG	4	234.75	231.75	-3	0.99	-1%
10	IG	5	623	608	-15	0.98	-2%
17	IG	5	726	721	-5	0.99	-1%
19	IG	5	598	588	-10	0.98	-2%
20	IG	1	21	21	0	1.00	0%
3	SG	5	508.25	494.75	-13.5	0.97	-3%
6	SG	2	326	315	-11	0.97	-3%
8	SG	5	1024	1027.25	3.25	1.00	0%
11	SG	5	980.5	974.5	-6	0.99	-1%
14	SG	1	748	748	0	1.00	0%
16	SG	3	184	181	-3	0.98	-2%
18	SG	5	854	1142	288	1.34	34%

Supplementary Table 1: Sum lesion score of the impact (IG) and stretching group (SG).

Week	Exercise	Exercise duration	Repetitions per exercise	Sets	Break	Jump height
1-4	Marching	30 sec	-	2	30-60 sec	-
	hoping (on the spot)	-	Up to 10 repetitions	3	30-60 sec	-
	Stepping up and down	30-60 sec	-	2	30-60 sec	Up to 15cm Up to 20cm
5-8	Stepping up, jumping off (and jumping up)	-	Up to 10-15 jumps	2	30-60 sec	Up to 15cm
	Hopping (on the spot and in motion)	-	Up to 15-20 repetitions	3	30-60 sec	-
	Jumping	-	Up to 10 jumps	2	30-60 sec	Up to 10cm
	Jumping high	-	Up to 10 jumps	2	30-60 sec	Up to 10cm
9-12	Jumping	-	Up to 10-15 jumps	3	30-60 sec	Up to 10cm
	High jump	-	Up to 10-15 jumps	3	30-60 sec	Up to 15cm
	Long jump	-	Up to 10 jumps	2	30-60 sec	Up to 20cm
13-16	High jump	-	Up to 10-15 jumps	3	30-60 sec	Up to 20cm
	Long jump	-	Up to 10-15 jumps	3	30-60 sec	Up to 30cm
	Jumping down	-	Up to 10 jumps	2	30-60 sec	Up to 15cm
17-20	High Jump	-	Up to 15 jumps	3	30-60 sec	Up to 25cm
	Long jump	-	Up to 15 jumps	3	30-60 sec	Up to 40cm
	Jumping down	-	Up to 10-15 jumps	3	30-60 sec	Up to 15cm
	1 legged jump	-	Up to 10 jumps per side	2	30-60 sec	Up to 20cm
21-24	high jump	-	Up to 15 jumps	3	30-60 sec	-
	long jump	-	Up to 15 jumps	3	30-60 sec	Up to 30cm
	downward jump	-	Up to 10-15 jumps	3	30-60 sec	Up to 40cm
	1-legged jump	-	Up to 10 jumps per side	3	30-60 sec	Up to 20cm

Supplementary Table 2: Training plan of impact exercise.

	Impact group (n=9)	Stretching group (n=7)
Adherence rate at average training duration		
Supervised training sessions	Ø 34.4 of 48 [23-46]	Ø 35.7 of 44 [26-41]
Home based training	Ø 13 of 24 [9-19]	-
Average training duration	30 minutes [25-40]	45 minutes
Number of supervised training sessions carried out		
At all	310	250
<100% of the training plan	32	-
100% of the training plan	135	-
>100-200% of the training plan	124	
>200% of the training plan	19	-
Average perceived load (1=very light -10=very hard)	Ø 6.63 [5-8.5]	-
Average well-being scale (1=very poor – 10=very good)	-	Ø 7.8 [5-10]
Numbers and reasons of absence		
Number of absences	Ø 12.4 [2-25]	Ø 7.7 [3-16]
Medical Reasons	68	21
Private Reasons	42	29
Study-related Reasons	2	4

Supplementary Table 3: Training documentation for impact and stretching group, Ø mean [min-max].

	**Impact group (n=9)**	**Stretching group (n=7)**
Organization		
1) How did you find the appointment process?	Ø 2 [1-3]	Ø 1.1 [1-2]
2) Do you associate any organizational effort with the training?	No: 1<br>Yes: 6<br>Missing data: 2	No: 4<br>Yes: 3
3) How long is your journey?	Ø 31km [4-400km]	Ø 24km [6-80 km]
4) Did you have to cancel one or more training sessions?	No: 0<br>Yes: 7<br>Missing data: 2	No: 0<br>Yes: 7
During the training session		
5) How did you perceive the load?<br>(1=very light – 10=very hard)	Ø 6 [2-9]	Ø 3.4 [2-7]
6) How did you find the duration of the training?<br>(1=too short – 10=too long)	Ø 5 [4-6]	Ø 3.8 [2-5]
7) How were the exercises explained?<br>(1=very understandable – 10=not understandable)	Ø 1.1 [1-2]	Ø 1.6 [1-4]
8) How did you find carrying out the exercises?<br>1=very easy – 10=not easy)	Ø 1.1 [1-5]	Ø 1.6 [1-8]
9) Were you able to complete the full training programme?	No: 2<br>Yes: 5<br>Missing data: 2	No: 1<br>Yes: 6
10) Did you have any pain during training?	No: 4<br>Yes: 3<br>Missing data: 2	No: 7<br>Yes: 0
11) How severe was the pain?<br>(1=not severe – 10=very severe)	Ø 3 [3]	No pain reported
12) What kind of pain have you experienced?<br>- bone pain<br>- muscle pain<br>- other pain	Bone pain: 2<br>Muscle pain: 0<br>Other pain: 1	No pain reported
After the training session		
13) How do you feel after training?<br>(1=very good – 10=not good)	Ø 2.4 [1-6]	Ø 3.1 [2-9]
14) Have you experienced any problems or complaints after training?	No: 5<br>Yes: 2<br>Missing data: 2	No: 7<br>Yes: 0

Supplementary Table 4: Results of the feasibility questionnaire of impact and stretching group, Ø, mean [min-max].

	Impact Group (n=9)					Stretching Group (n=7)				
	Scales	Baseline	6 months	Absolute Change	P Value	Scales	Baseline	6 months	Absolute Change	P Value
EORTC QLQ C30	Global health Status					Global health Status				
	Global health status	66.7 [37.5;79.2]	83.3 [58.3;95.8]	16.7 [4.2;29.2]	0.020*	Global health status	50.0 [41.7;58.3]	66.7 [50.0;75.0]	8.3 [0.0;25.0]	0.140
	Functional Scales					Functional Scales				
	Functional Scale	86.7 [63.3;88.9]	80.0 [60.0;87.8]	-2.2 [-12.2;3.3]	0.398	Functional Scale	58.9 [46.7;64.4]	77.7 [53.3;80.0]	8.9 [4.4;18.9]	0.018*
	Physical functioning	80.0 [80.0;96.7]	86.7 [80.0;87.8]	0.0 [-3.3;10.0]	0.683	Physical functioning	70.0 [46.7;80.0]	80.0 [53.3;86.7]	6.7 [3.3;13.3]	0.026*
	Role functioning	66.7 [41.7;100]	66.7 [66.7;100]	0.0 [-8.3;33.3]	0.396	Role functioning	33.3 [16.7;33.3]	66.7 [16.7;66.7]	16.7 [-8.3;33.3]	0.026*
	Emotional functioning	75.0 [66.7;87.5]	66.7 [58.3;83.3]	-8.3 [-16.7;4.1]	0.263	Emotional functioning	66.7 [41.7;91.7]	91.7 [58.3;100.0]	25.0 [8.3;33.3]	0.051
	Cognitive functioning	100 [66.7;100]	66.7 [50;100]	0.0 [-16.7;0.0]	0.059	Cognitive functioning	66.7 [50;83.3]	66.7 [50;83.3]	0.0 [-16.7;0.0]	0.564
	Social functioning	100 [66.7;100]	66.7 [50;100]	0.0 [-16.7;0.0]	0.059	Social functioning	66.7 [50;83.3]	66.7 [50;83.3]	0.0 [-16.7;0.0]	0.564

Supplementary Table 5: Results of the quality of life assessment using the EORTC QLQ-C30. Wilcoxon test, median [interquartile range], *nominal P<0.05 without correction for testing multiple endpoints.

Impact Group (n=9)						Stretching Group (n=7)					
	**Scales**	**Baseline**	**6 months**	**Absolute Change**	**P Value**		**Scales**	**Baseline**	**6 months**	**Absolute Change**	**P Value**
**EORTC QLQ C30**	Symptom Scales					**EORTC QLQ C30**	Symptom Scales				
	Symptom Scale	20.5 [10.3;30.8]	20.5 [6.4;38.4]	2.5 [-6.4;2.6]	0.905		Symptom Scale	28.2 [23.1;41.0]	23.1 [12.8;28.2]	-5.1 [-14.1; -5.1]	0.173
	Fatigue	33.3 [11.1;38.9]	22.2 [5.6;61.1]	0.0 [-11.1;11.1]	0.892		Fatigue	77.8 [44.4;83.3]	44.4 [33.3;44.4]	-11.1 [-38.9;0.0]	0.140
	Nausea and vomiting	0 [0.16.7]	0 [0;8.3]	0 [-8.3;8.3]	1.000		Nausea and vomiting	0 [0;0]	0 [0;33.3]	0.0 [-16.7;0.0]	0.180
	Pain	33.3 [0;50]	33.3 [16.7;50]	0 [0.0;16.7]	0.257		Pain	50.0 [33.3;66.7]	33.3 [0;66.7]	-16.7 [-33.3;0.0]	0.066
	Dyspnea	33.3 [0;50]	33.3 [0;50]	0 [0.0;0.0]	1.000		Dyspnea	33.3 [33.3;66.7]	33.3 [0;66.7]	0.0 [-33.3;0.0]	0.317
	Insomnia	33.3 [0;66.7]	33.3 [33.3;50]	0 [-16.7;33.3]	0.414		Insomnia	0 [0;33.3]	0 [0;33.3]	0.0 [-33.3;0.0]	0.157
	Appetite loss	0 [0;33.3]	0 [0;16.7]	0 [0.0;0.0]	0.317		Appetite loss	0 [0;0]	0 [0;0]	0.0 [0.0;0.0]	0.317
	Constipation	0 [0;0]	0 [0;33.3]	0.0 [0.0;16.7]	0.564		Constipation	0 [0;33.3]	0 [0;66.7]	0.0 [0.0;33.3]	0.564
	Diarrhoea	0 [0;33.3]	0 [0;33.3]	0.0 [0.0; -33.3]	0.655		Diarrhoea	0 [0;33.3]	0 [0;66.7]	0.0 [0.0;33.3]	0.414
	Financial difficulties	33.3 [0;33.3]	0 [0;33.3]	0.0 [0.0]	0.317		Financial difficulties	0 [0;0]	0 [0;33.3]	0.0 [0.0;33.3]	0.180
	Summary	86.7 [64.7;91.7]	75.0 [65.7;93.1]	-1.8 [-9.1;4.5]	0.767		Summary	68.2 [58.5;72.6]	80.7 [65.4;81.7]	7.8 [4.6;13.5]	0.043*

Supplementary Table 6: Results of the quality of life assessment using the EORTC QLQ-C30, Wilcoxon test, median [interquartile range], *nominal P<0.05 without correction for testing multiple endpoints.

	Impact Group (n=9)						Stretching Group (n=7)				
	Scales	Baseline	6 months	Absolute Change	P Value		Scales	Baseline	6 months	Absolute Change	P Value
EORTC QLQ MY20	Symptom Scales					EORTC QLQ MY20	Symptom Scales				
	Symptom Scales	25.0 [12.2;43.3]	29.2 [10.6;54.2]	0.0 [-10.0;11.2]	0.779		Symptom Scales	26.6 [15.5;25.8]	16.7 [12.5;27.0]	-12.5 [-14.1; -3.9]	0.042*
	Disease symptoms	27.8 [5.6;48.9]	22.2 [11.1;66.7]	0.0 [-8.3;22.2]	0.481		Disease symptoms	27.8 [16.7;50.0]	16.6 [0.0;33.3]	-11.1 [-16.7;0.0]	0.102
	Side Effect of Treatment	23.3 [17.6;41.7]	25.5 [9.7;46.3]	1.8 [-10.2;7.9]	0.515		Side Effect of Treatment	29.6 [14.8;40.0]	21.0 [11.1;22.2]	-11.1 [-18.1; -6.9]	0.028*
	Functional Scales						Functional Scales				
	Functional Scales	58.3 [45.8;70.8]	58.3 [37.5;66.7]	0.0 [-16.7;4.2]	0.196		Functional Scales	66.7 [41.7;91.7]	58.3 [50.0;100.0]	8.3 [-16.7; 8.3]	0.729
	Body Image	66.7 [33.3;100]	66.7 [33.3;100]	0.0 [-16.7;0.0]	1.000		Body Image	66.7 [33.3;100]	100 [66.7;100]	33.3 [0.0;33.3]	0.180
	Future Perspective	55.5 [44.4;72.2]	55.5 [38.9;72.2]	-11.1 [-27.8;11.1]	0.203		Future Perspective	66.7 [44.4;88.9]	55.6 [44.4;100]	0.0 [-11.1;11.1]	0.705

Supplementary Table 7: Results of the quality-of-life assessment using the disease-specific questionnaire EORTC QLQ-MY20, Wilcoxon test, median [interquartile range], *nominal P<0.05 without correction for testing multiple endpoints.

		Impact Group (n=9)				Stretching Group (n=7)			
Variable	Normal range	Baseline	6 months	Change in %	P Value	Baseline	6 months	Change in %	P Value
Hb [g/dl]	13.5-16-9 (m), 12-16 (f)	12.1 [11.1; 12.7]	12.4 [11.9; 13.95]	+2.5	0.07	12.6 [12.1; 14.5]	12 [11.6; 15.1]	-4.8	0.469
Calcium [mmol/l]	2.0-2.7	2.3 [2,25; 2,4]	2,3 [2,2; 2,4]	0	1	2,4 [2,3; 2,4]	2,4 [2,2; 2,4]	0	1
Creatinine [mg/dl]	0-1.17 (m), 0-0.95 (f)	0.77 [0.71; 0.85]	0.73 [0.68; 0.87]	-5.2	1	1.15 [0.98; 1.26]	1.2 [1.04; 1.24]	+4.3	0.844
Vitamin D [µg/l]	20-70	28.8 [24.55; 34.3]	26.8 [21.3; 31.5]	-6.9	0.172	43.8 [22; 60.6]	38.2 [16.9; 57.2]	-12.8	0.047*

Supplementary Table 8: Serum levels of blood parameters, median [interquartile range].
